# Supplementary material for: Tracking the evolution of esophageal squamous cell carcinoma under dynamic immune selection by multi-omics sequencing
Source: Nat Commun. 2023 Feb 17;14:892. doi: 10.1038/s41467-023-36558-1 (PMC9938262; doi:10.1038/s41467-023-36558-1)
Supplement: Supplementary file 1 — Supplementary Figures [file 41467_2023_36558_MOESM1_ESM.pdf]

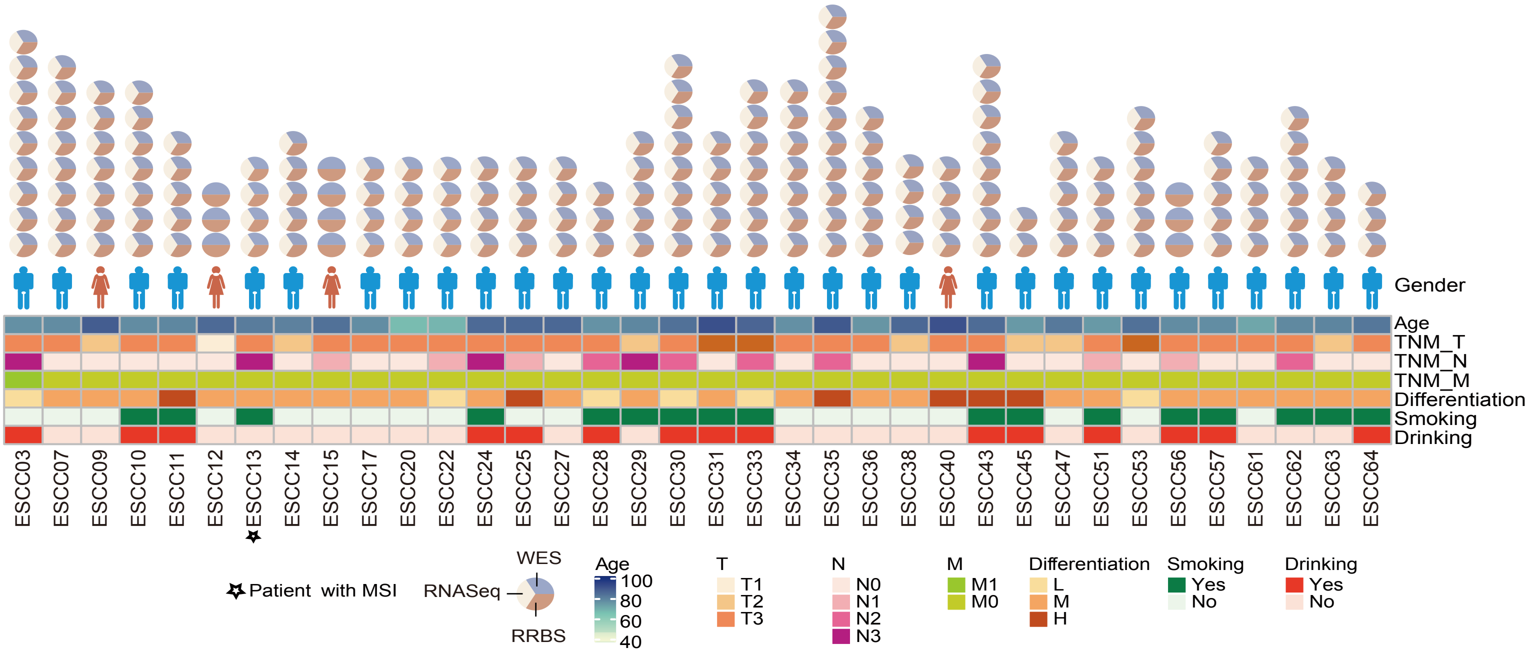

Supplementary Fig. S1. The landscape of clinical characteristics of the ESCC patients in the study.

The pie charts in the top panel show the sequencing strategies for each sample. The number of pies corresponds to the number of sampling tumor sites. All ESCC patients' clinical and pathological information was represented on the bottom, including gender (middle panel), age, smoking status, drinking history, classic pathological tumor stage, and tumor differentiation.

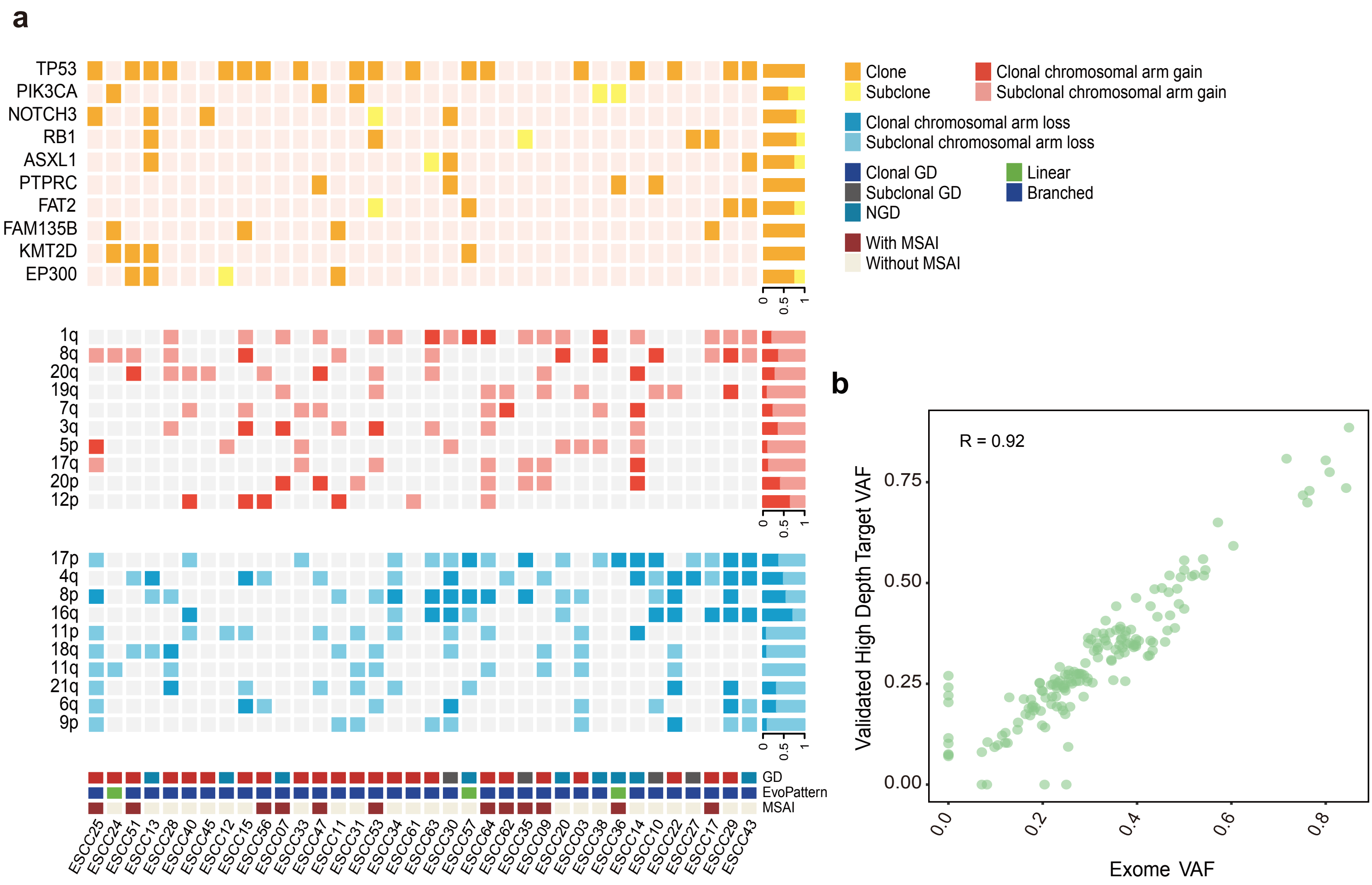

Supplementary Fig. S2 Genomic alterations in ESCC.

a Heatmap shows the clonal status of somatic mutations and arm level somatic copy number alterations.

b Correlation of variant allele frequencies obtained from exome sequencing and high depth target VAF.

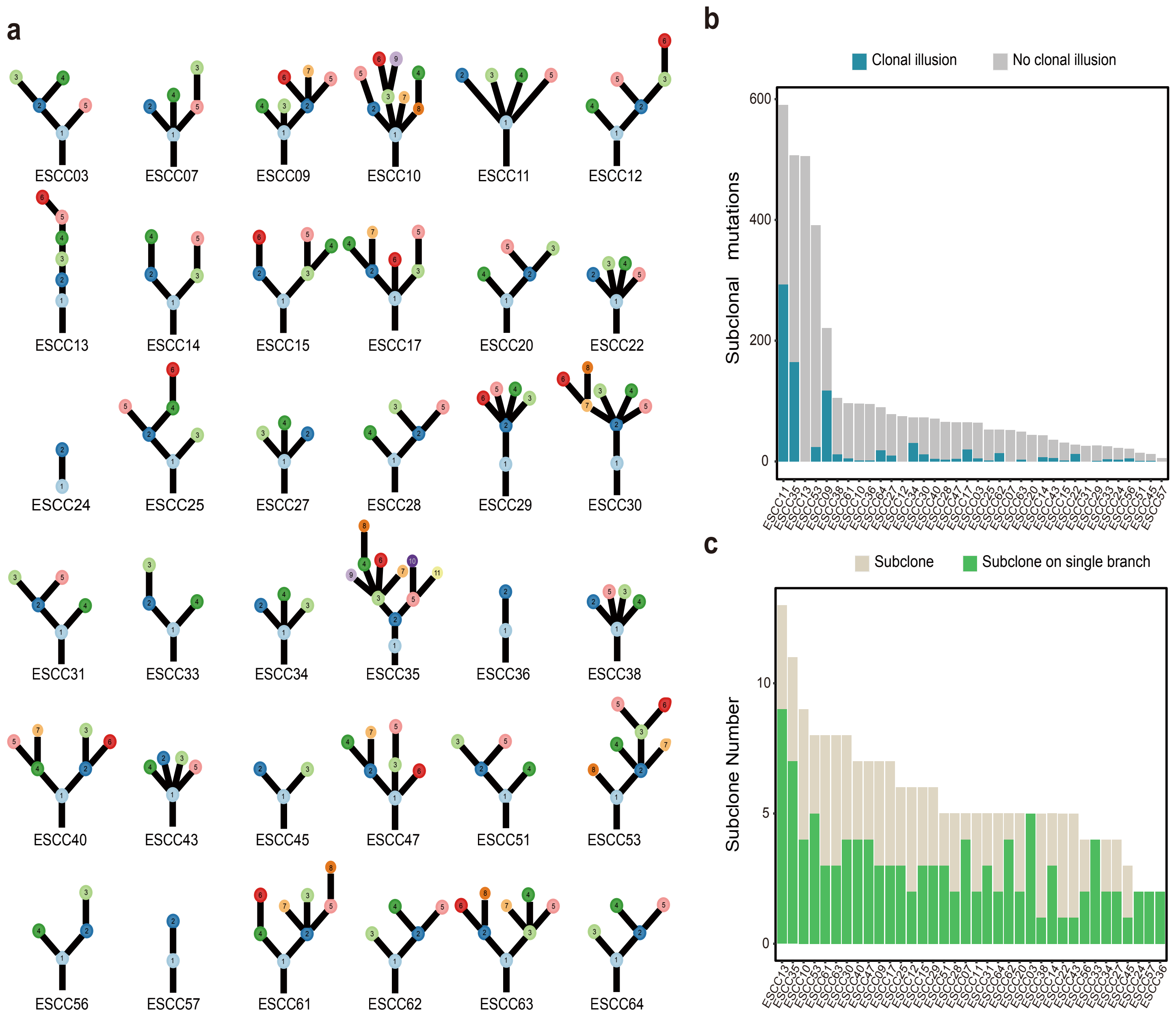

Supplementary Fig. S3 Clonal evolution maps of ESCC patients.

a The inferred clonal evolution trees of 36 ESCC patients.

b The subclonal illusion barplot of each patient was shown, the blue bar represent clonal illusion.

c Subclone number for each patient, the green bar represents the subclone on single branch.

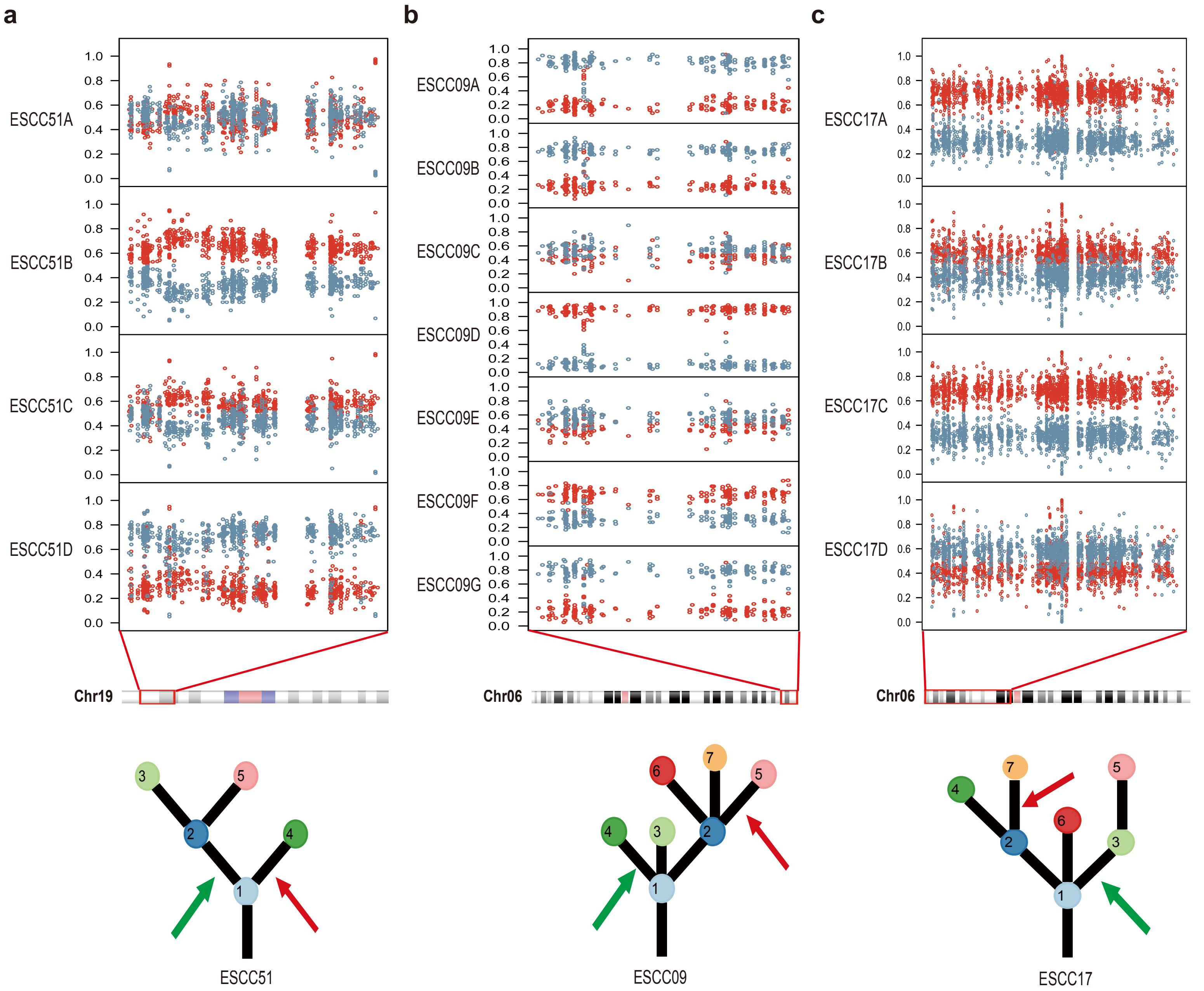

Supplementary Fig. S4 Mirrored subclonal alleleic imbalance events across ESCC cohorts.
a-c LogR and B allele frequency of heterozygous SNPs across all tumor regions from the whole chromosomes. The mirrored subclonal allelic imbalance was captured in the three representative samples (ESCC51, ESCC09 and ESCC17). Phylogenetic trees are indicated the parallel evolution of amplifications and mirrored subclonal allelic imbalance (arrows).

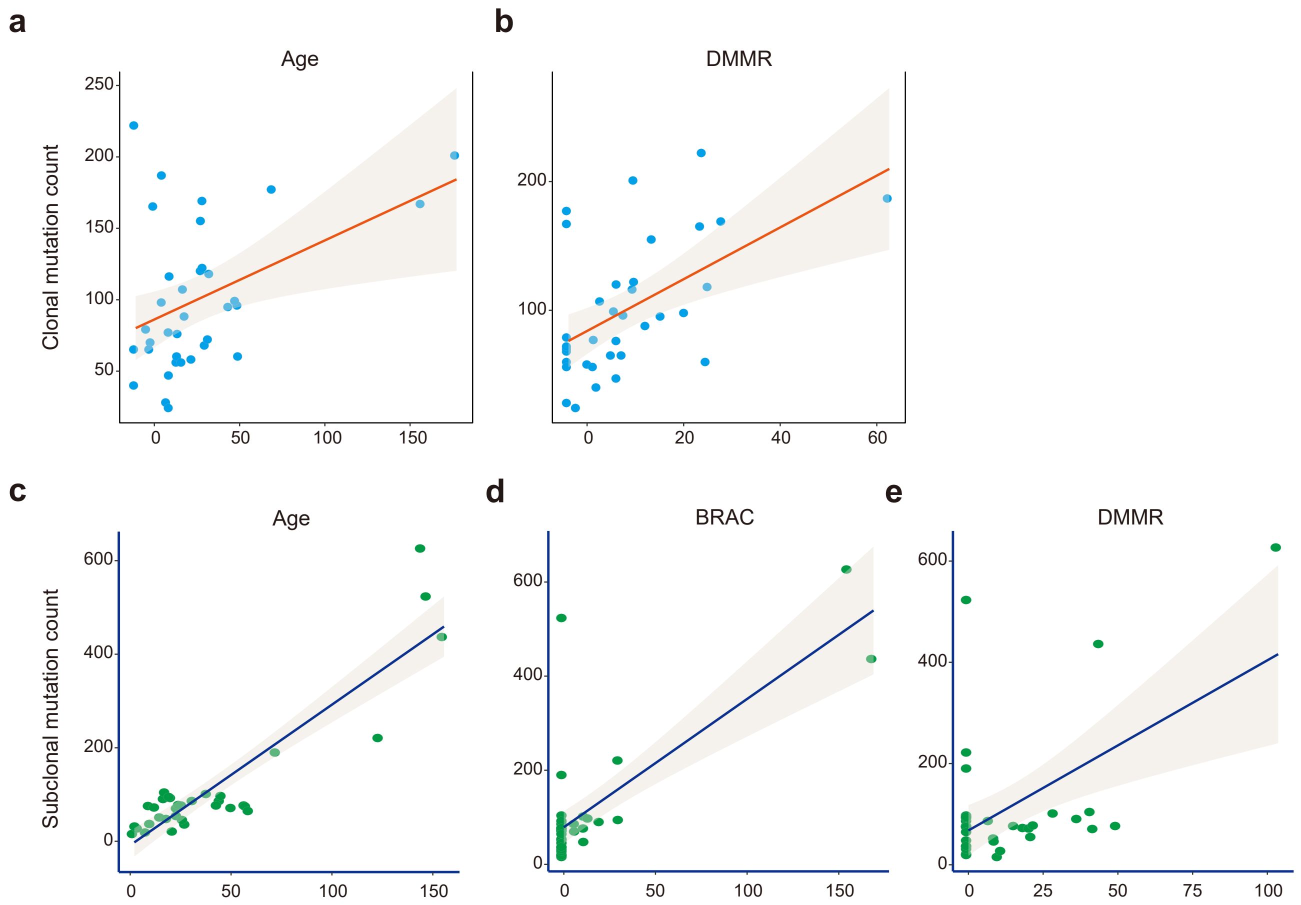

Supplementary Fig. S5 Key driving events of mutation signature.

a-b Correlation of prevalence of mutational signatures (Age and DMMR) and clonal mutation count in the ESCC cohort.

c-e Correlation of prevalence of mutational signatures (Age, BRAC and DMMR) and subclonal mutation count in the ESCC cohort. Line of best fit shown in red/blue and gray area represents 95% confidence bands.

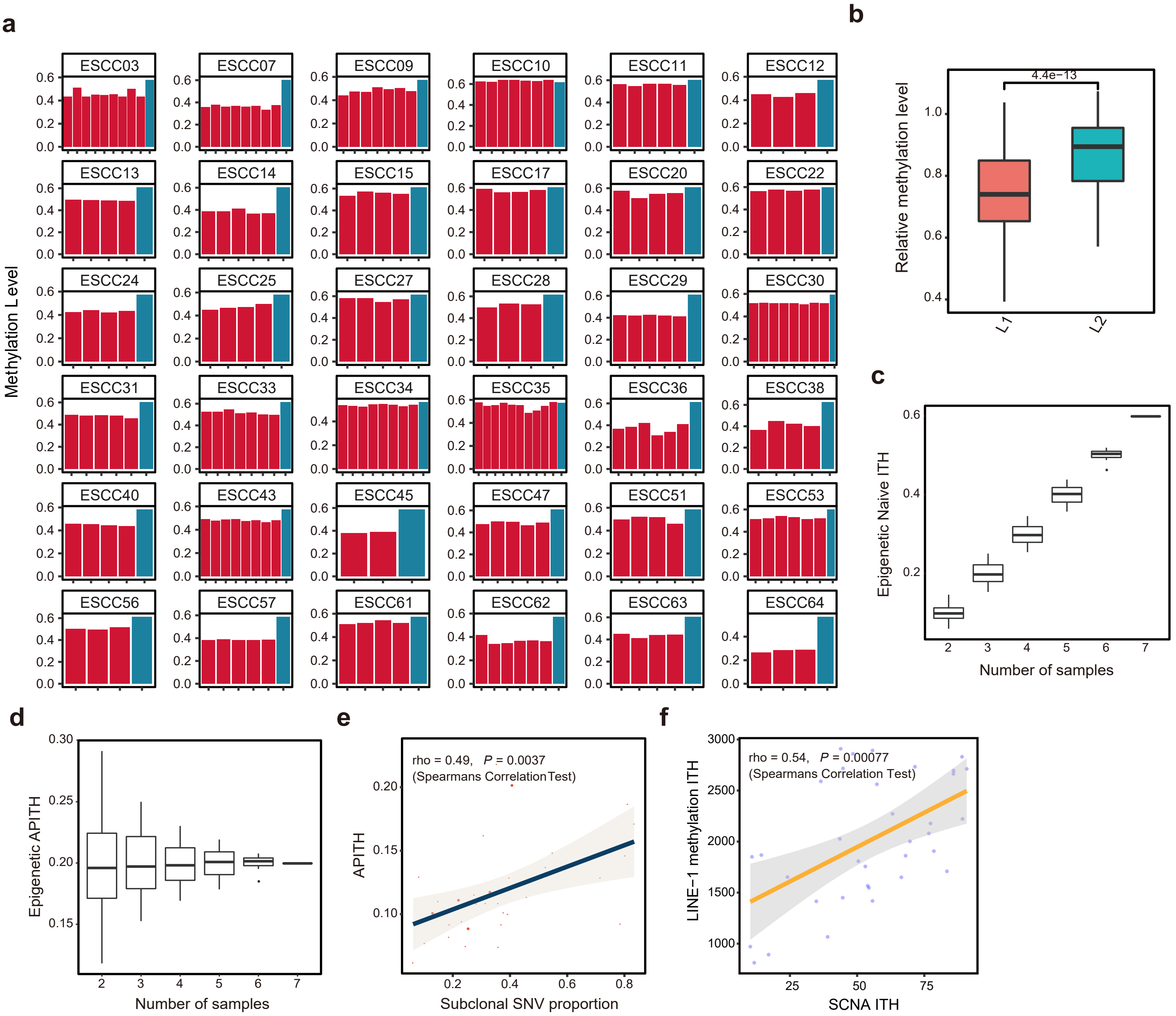

Supplementary Fig. S6 The global hypomethylation trend in ESCC tumor.

a Global methylation levels (1-kb bin) of each sample for patients (n = 36). The red bar represents tumor samples, and the blue bar represents paired normal tissue.

b Compared with paired normal tissue (n = 36), the relative DNA methylation levels of L1 and L2 in tumor tissue (n = 186). The minimum and maximum are indicated by the extremes of the box plot; the median is indicated by the thick horizontal line; and the first and third quartiles are indicated by box edges; two-sided Wilcoxon rank-sum test.

c-d Methylation level APITH score (**d**) and naïve (**c**) calculated based on the tumor sites of ESCC09 (n = 7). APITH score was calculated as the average pairwise distance between any pair of tumor samples. The minimum and maximum are indicated by the extremes of the box plot; the median is indicated by the thick horizontal line; and the first and third quartiles are indicated by box edges.

e Subclonal SNV proportion correlated with epigenetic APITH. The scatter plot shows the Spearman correlation between patient-wise SNV-ITH and patient-wise epigenetic ITH in the ESCC cohort (n = 36 patients).

f Scatter plot represents the Spearman correlation between LINE-1 methylation ITH and SCNA ITH in the ESCC cohort (n = 36 patients). Spearman's correlation coefficient (rho) and corresponding P-value is shown. All data are presented as mean values +/SEM. Line of best fit shown in dark blue/orange and gray area represents 95% confidence bands.

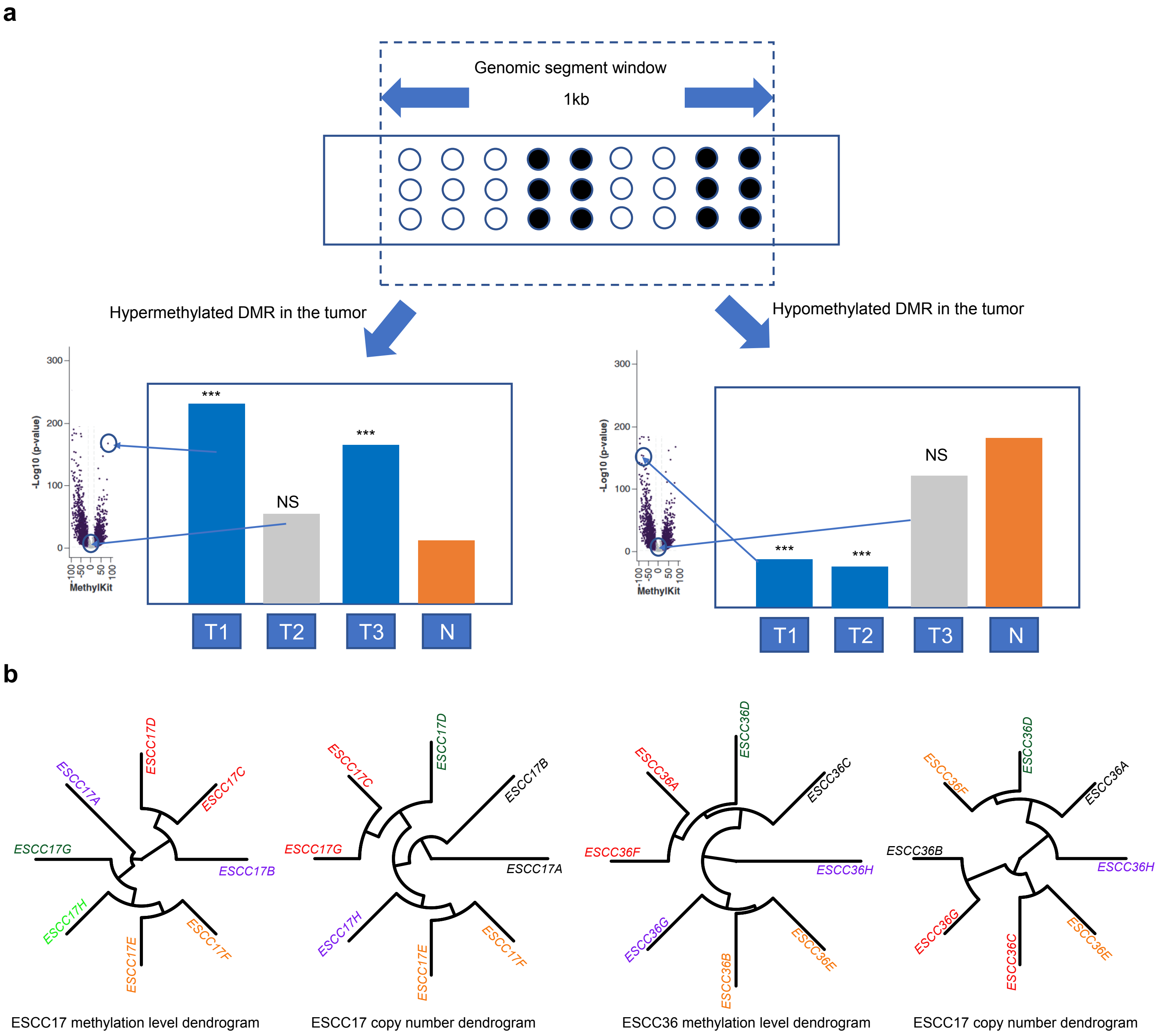

Supplementary Fig. S7 Phylogenetic and epigenetic trees construction based on the methylation level of DMR.

a Schema exhibits the methylation level of intratumoral heterogeneity using the DMR. The bar in blue represented the significant differential methylated regions (DMR) and the bar in grey represented the non-significant regions. We got all the DMR regions to evaluate the methylation ITH of each patient using the APITH method.

b Phylogenetic and phyloepigenetic trees were constructed using ESCC17 and ESCC36.

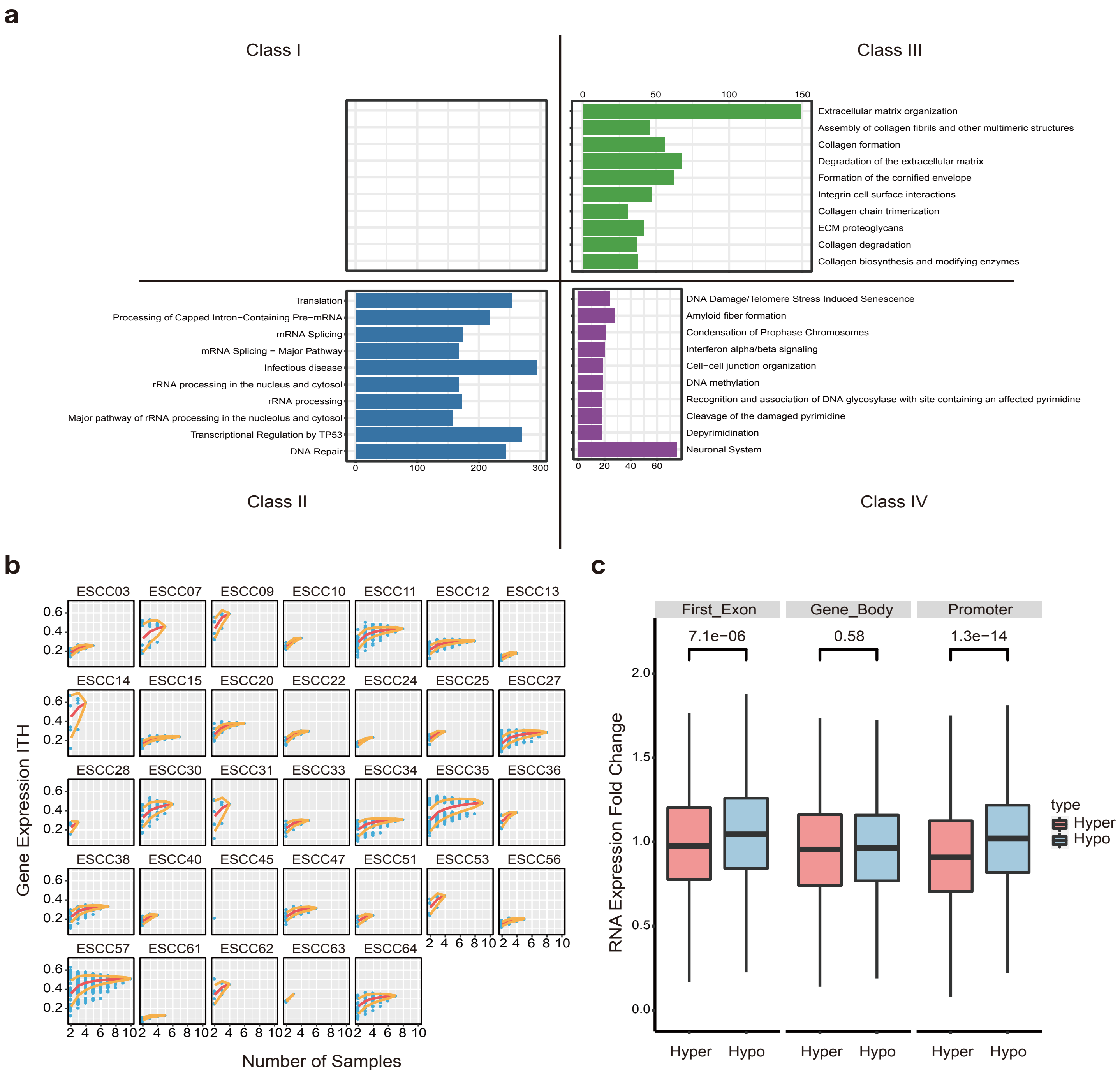

Supplementary Fig. S8 RNA heterogeneity in ESCC.

a The top 10 Reactome pathways for each RNA heterogeneity class barplot are shown: (Class I, Class II, Class III, Class IV).
b RNA-ITH scores were evaluated from each tumor by sampling one to N biopsies (where N is the total number of biopsies) in our ESCC cohort (n = 33). The RNA-ITH score (y-axis) is shown for all possible subgroups of tumor sites.
The mean (red line) and standard deviation (orange lines) are plotted for each patient.
c The boxplot shows the association between differential methylation status and gene expression between different tumor sites, stratified by genomic features: gene promoters, first exons, and gene body (excluding the first exon) in our ESCC cohort (n = 33). The minimum and maximum are indicated by the extremes of the box plot; the median is indicated by the thick horizontal line; and the first and third quartiles are indicated by box edges; *P*-value is shown; two-sided Wilcoxon rank-sum test.

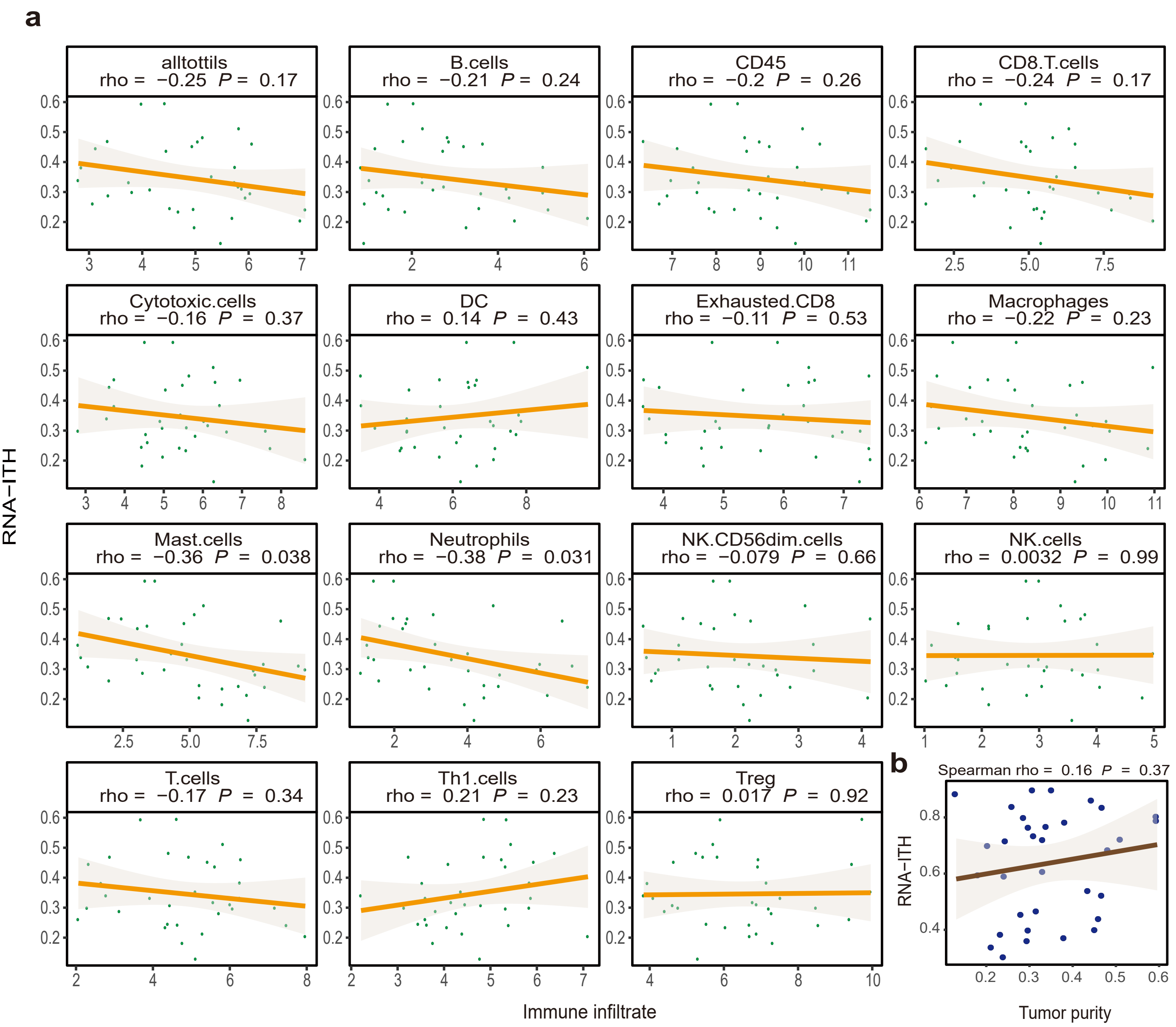

Supplementary Fig. S9 Correlation between RNA-ITH scores and different immune cells.

a Spearman correlation between patient-level RNA-ITH scores and immune infiltration measurements from RNAseq using the Danaher method.

b The scatter plot represents the correlation between patient-wise RNA-ITH scores and tumor purity from the WES data using Sequenza (n = 33 ESCC patients).

Spearman's correlation coefficient (rho) and corresponding P-value is shown. All data are presented as mean values +/- SEM. Line of best fit shown in orange/brown and gray area represents 95% confidence bands

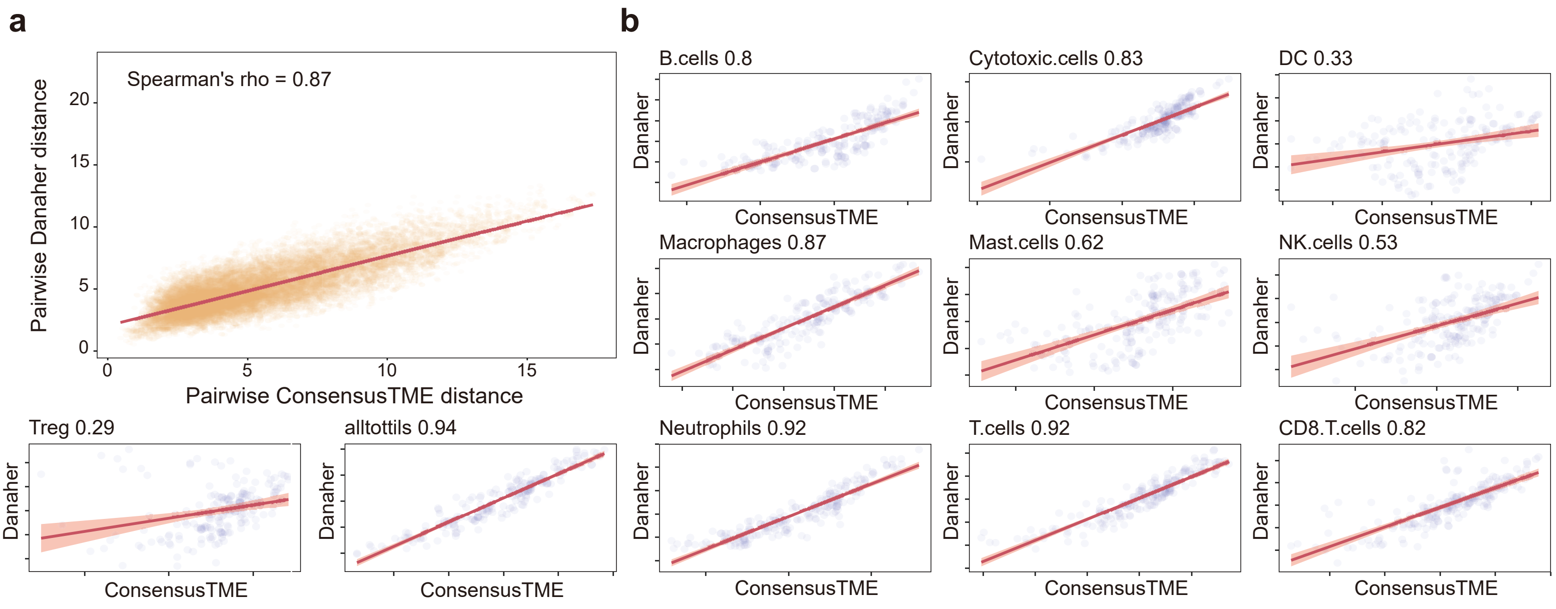

Supplementary Fig. S10 Comparation between ConsensusTME and Danaher methods.

a The pairwise immune distances between every two tumor regions from the same patient using Consensus^{TME} and Danaher methods are compared for the ESCC cohort with RNA-seq data.

b The correlation between Danaher and ConsensusTME across different immune cell types.

Line of best fit shown in dark red and light red area represents 95% confidence bands. All data are presented as mean values +/- SEM.

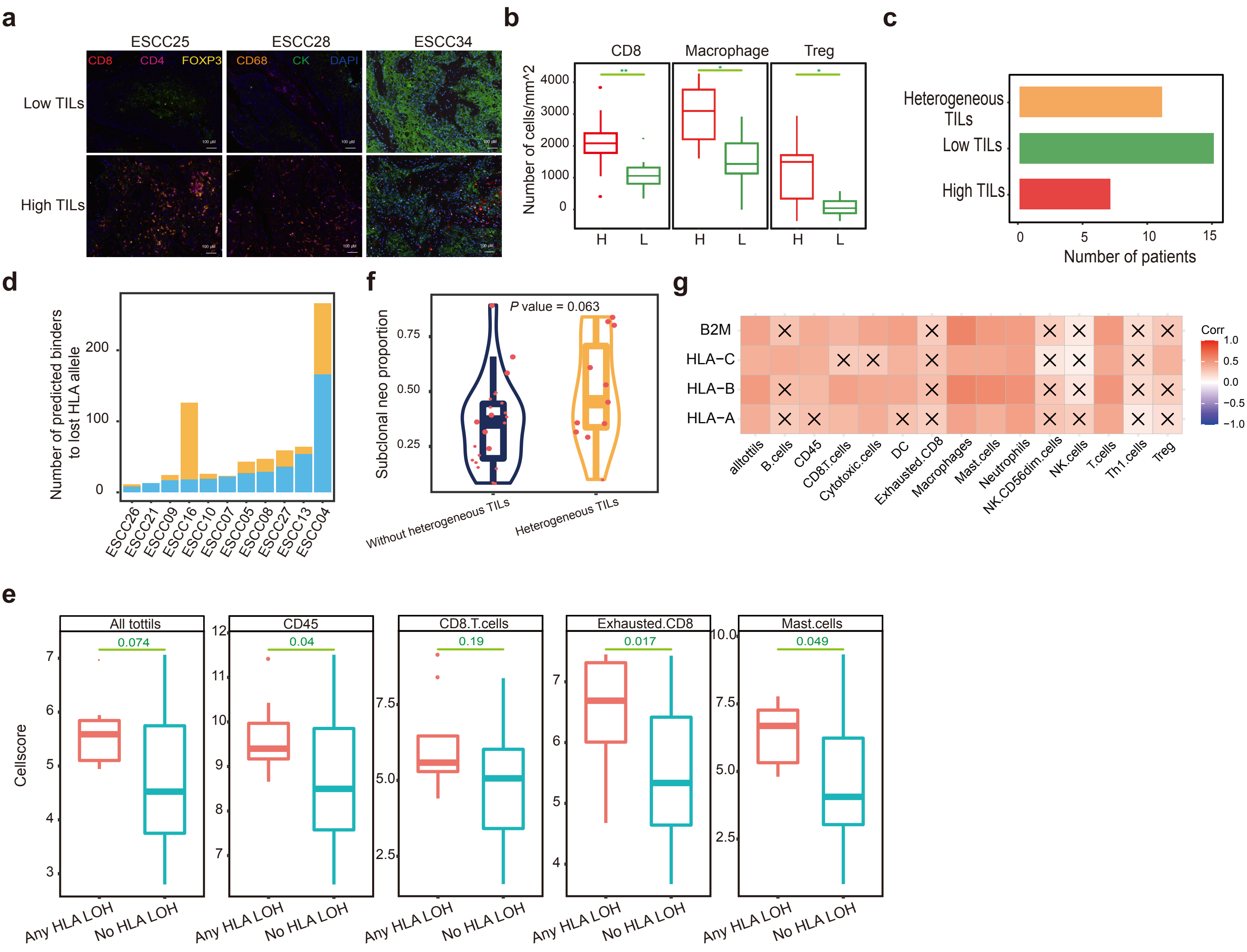

Supplementary Fig. S11 Immune evasion events in ESCC.

a Multiplex immune-staining of representative patients (ESCC25, ESCC28, and ESCC34) with heterogeneous TILs. Scale bar is shown (100 μm). Date are repeated once for each patient's tumor samples.

b Boxplot shows the number of immune cells between High (H, n = 7) and Low (L, n = 15) TILs samples. The minimum and maximum are indicated by the extreme points of the box plot; the median is indicated by the thick horizontal line; and the first and third quartiles are indicated by box edges; * $P < 0.05$,** $P < 0.01$; one-sided Student t test .

c The bar plot shows the number of patients in different immune infiltration group.

d The bar plot shows the total number of mutations predicted to bind to the lost allele for all ESCC (n = 11). The mutation clonal status is also indicated as clonal (dark blue) or subclonal (orange).

e Expression of lymphocyte markers in cases with no HLA LOH (n = 25) or any HLA LOH (n = 11). The minimum and maximum are indicated by the extreme points of the box plot; the median is indicated by the thick horizontal line; and the first and third quartiles are indicated by box edges; P-value is shown; two-sided Wilcoxon rank-sum test.

f Violin boxplot displays subclonal neo proportion for each patient is shown, grouped by immune classification: without Heterogeneous TILs (n = 22) and Heterogeneous TILs (n = 11). The minimum and maximum are indicated by the extreme points of the box plot; the median is indicated by the thick horizontal line; and the first and third quartiles are indicated by box edges; P-value is shown; two-sided Wilcoxon rank-sum test.

g Heatmap shows correlation z-transformed values of HLA type, B2M gene and immune cell infiltration. The cell of the heatmap without a cross indicated significant correlation between the corresponding gene expression and immune cell infiltration.

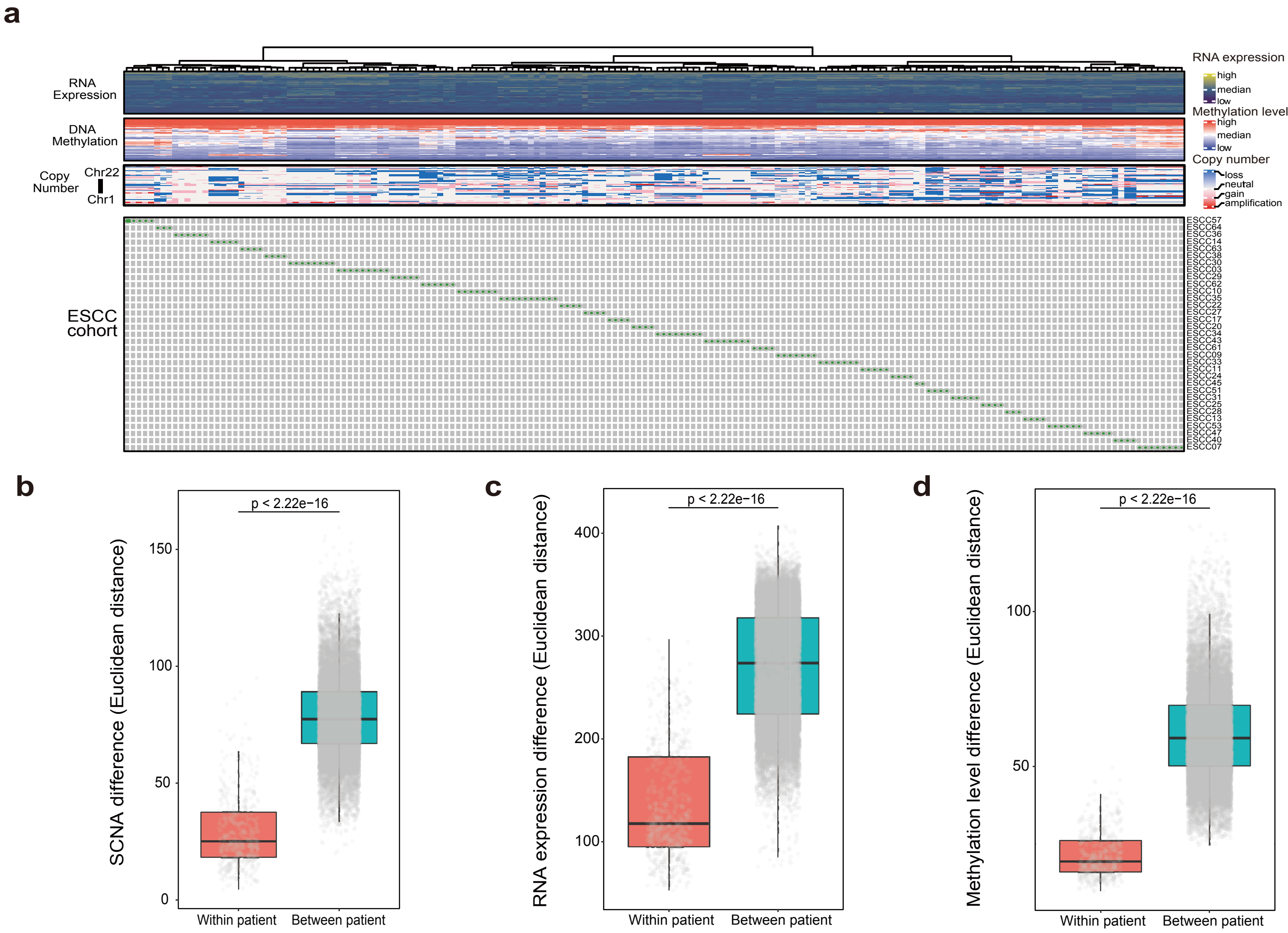

Supplementary Fig. S12 Integrative analysis of triomics in ESCC

a The dendrogram and three colored heatmaps (top) represent the hierarchical clustering of tumor regions (columns) in the ESCC cohort (33 patients, 176 tumor region samples) based on the copy-number profile, RNA-expression, and methylation levels. The first heatmap shows the top 300 variably expressed genes (rows). The second heatmap shows the top 500 variable methylation level bins. The third heatmap shows the copy number profiles of each tumor sample. The sparse heatmap (bottom) shows tumor regions per patient (rows).

b-d Euclidean distance of SCNA differences (**b**), RNA expression differences (**c**), and Methylation level differences (**d**) across 33 patients with 176 tumor region samples. Dots correspond to sample pairs in the same (left) and different (right) patients. The minimum and maximum are indicated by the extreme points of the box plot; the median is indicated by the thick horizontal line; and the first and third quartiles are indicated by box edges; P-value is shown; two-sided Wilcoxon rank-sum test.
